# Medical and Physical Intelligence

**Published:** 1824-04

**Authors:** 


### MEDICAL AND PHYSICAL INTELLIGENCE.

### PATHOLOGY.

1. *Table of Organic Changes resulting from Inflammation.*—M. VILLERME has recently published the following arrangement of the morbid effects of inflammatory action, which he conceives to be more methodical, and more in conformity with their natural connexion and successive development, than any hitherto contrived.

I. *Lesions, or immediate alterations.*—Unusual accumulation of blood in the minute vessels; redness and tumefaction of the inflamed parts; greater consistence of those which are soft.

II. *Lesions, or organic changes, which are terminations of inflammation.*—Augmentation of volume; hardening; hepatization; dropsy; serous infiltration; opacity of tissues which are naturally transparent; suppuration; false membranes; purulent infiltration; purulent drains; abscess; deposition by congestion; vesication; softening; fleshy excrescences; narrowing and obstruction of vessels and canals; adhesions, 1st, between surfaces naturally free (serous and mucous), obliteration of serous cavities, bridles or bands traversing them; obstructions; 2d, adhesion between accidental surfaces; cicatrization by the first intention; cicatrization by the second intention, with or without destruction of parts, (false membranes.) Erosion, or ulceration; perforation; gangrene; hospital gangrene; schirrus; fungus hæmatodes; some other morbid structures not yet well understood. Cancer; tubercles,

1st, scrofulous, 2d, of serous membranes. Accidental tissues, having something analogous in the economy, cartilaginous and cartilagineform *(cartilageniforme)*, osseous and ossiform *(ossiforme)*; fibrous and resembling fibrous, (false joints;) mucous (membrane of fistulous openings); dermoid, serous, (cysts); cellular; particular monstrosities; congenital acclusions, &c. Sanguiferous vessels of new formation or creation; organization of the fibrin of the blood, of pus, of false membranes, &c. and conversion into laminated, serous, &c. structures; accidental organs, apoplectic cysts, &c. membranous canals of fistules; synovial capsules of false articulations.

III. *Changes which are the remote consequences of inflammation, and which are not effected until this has already ceased.*—Return of parts to their natural state: restoration of obliterated cavities; restoration of the obliterated medullary cavity of a bone; reproduction of the marrow and the medullary organ; return of canals and vessels to their ordinary dimensions; reproduction of fat.

It is sufficient, (adds M. Villermé,) when we have a tolerably profound knowledge of pathology, to glance at this table, in order to perceive how numerous and varied are the organic changes of structure which constitute the subject of this article. It will be at once recognised that there are some so intimately allied to inflammation, that we cannot imagine this to exist without them; others, accompany inflammations, marking their intensity, their duration, specific character, their site and particular tendency, &c.; while others are not to be found until the inflammatory action has passed away. The first are the inflammation itself; the second mark, by their formation, the actual existence of the inflammation; and the third show that it has ceased. *(Jour. General,* Decembre.)

2. *Aneurism of the Aorta opening into the Trachea.*—M. ANDRAL, jun. regards this case as remarkable; inasmuch as there was no aneurismal sac, properly speaking, but a mere augmentation in the capacity of the artery, to such an extent as to admit the fist; and, at the same time, a remarkable increase in the thickness of its coats. It was by a kind of ulceration of the tunics that the perforation of the vessel appeared to have taken place. This dilatation had not been attended with any remarkable symptom during life, excepting an indefinable rattling *(bruissement)* towards the upper part of the sternum. The patient perished by a tremendous hemorrhage. There was also, in this case, a considerable emphysema of the lung on one side; the existence of which M. Andral recognised during life, by the signs indicated by M. Laennec.—*(Revue Medicale,* Decembre.)

---

### THERAPEUTICS AND MATERIA MEDICA.

3. *Observations on the Use of Iodine in Medicine.*—Dr. J. G. KOLLEY, physician at Breslau, has published some interesting remarks on this subject; the results of his experience have led him to the following conclusions:—

1. Iodine, in the form of tincture, prepared after the formula of M.

Coindet, appears to exert a deleterious influence on the nervous system. It gives rise, in persons of sensible habits, to disagreeable accidents, even in doses of a few drops: these symptoms may degenerate into violent spasms. Such persons experience, after a few doses, a degree of restlessness in their limbs, which prevents them from remaining in the same position. The result of this is a feeling of lassitude and weight; violent head-ache, tremors, depression, moroseness, spasm, paleness of the countenance, tears, and great anxiety of the chest. They frequently have a particular dislike to the medicine. As to the rest, these symptoms are entirely different from those proceeding from their being saturated with the iodine.

2. Persons who are disposed to congestions about the head, or any other important organ, who are of robust constitution, muscular frame, well fed, and with the appearance of the bilious temperament, bear iodine ill,—at least, if purgatives are not exhibited at the same time. In these the congestions increase; the head becomes deranged, and cephalalgia comes on to a violent degree.

3. Whenever the digestive organs are not perfectly healthy, having a disposition to cardialgia and colic, the iodine must be given with much caution; otherwise, severe dyspepsia, with violent pain of the stomach, is apt to be induced.

4. All febrile and inflammatory affections absolutely prohibit the use of iodine, be they local or general.

5. The same holds good with regard to the disposition to diarrhœa, local affections of the urinary organs, *phthisis pulmonalis*, local affections of the lungs, stomach, liver; and, generally speaking, all those diseases of internal organs which are liable to terminate in suppuration. In persons who live on very nutritive diet, and who drink much wine, the iodine often excites fever and hemorrhage. Hectic diseases likewise contra-indicate its employment.

Iodine, on the other hand, is indicated—

1. In those patients who exhibit no symptoms of an excess of sensibility.

2. Where there are no active congestions, and where the venous system does not predominate.

3. When the digestive system is sound.

4. When there is no fever, nor tendency to diarrhœa, nor any of those morbid conditions specified above; and when the patient is well nourished without overloading the stomach.

The observations of Dr. Kolley incline him to the internal use of iodine, in the form of tincture; in which way it has appeared to him very powerful in the cure of goitre and scrofula. To insure its success in the former of these, it is recommended to ascertain the following circumstances:—1st. That the swelling really exists in the thyroid gland. 2d. That there is not too considerable a degeneration; that the goitre is neither schirrous nor carcinomatous; and that it does not contain either calcareous concretions or similar productions. 3d. That the tumor is not of too long standing. 4th. That there is neither cachexy nor hectic fever, nor saburræ in the primæ viæ, nor any alteration in the mass of the fluids. 5th. That the goitre itself be not inflamed and painful.—(*Archiv für Mediziniche Erfahrung.*)

4. Dr. Kolley's *Case of Goitre in his own Person, treated with Iodine.*—Scarcely had the account of this medicine been published, when Dr. Kolley resolved to try it for a goitre of considerable size, and of ten years' standing, which had already resisted all the supposed remedies of this disease. His respiration was much impeded; he could neither walk quickly, nor for any considerable length of time; he could not go up stairs without difficulty, nor exert his voice. To these symptoms were added frequent and violent congestions about the head. During the employment of the iodine, he scrupulously followed the prescription of M. Coindet (ten drops of the tincture three times a-day in syrup,) for a fortnight: this dose was taken without any inconvenience. During the second fortnight, he experienced disagreeable effects from the increase of the gland; his respiration became very much oppressed, his voice hoarse, the tumor indolent, the congestions violent; he had an unpleasant taste in the mouth, and his appetite fell off. He did not perceive that the excretions were changed. Next fortnight the gland diminished, and disappeared almost entirely in the space of twelve weeks, during which he continued the medicine without intermission. The lower lobe of the right side alone retained some degree of hardness, and the difficulty of breathing disappeared as much as the conformation of his chest permitted. He did not perceive any increase, either in the alvine evacuations, the urine, or perspiration; but he had diarrhœa during some days, having taken forty drops of the tincture at a time. He had no pain at the stomach, though his appetite was rather impaired; and he had frequent head-ache, which he attributed to congestion. In short, though he continued the use of iodine for three months, he perceived none of the symptoms attributed by M. Coindet to what he has called the period of saturation. He did not lose flesh.—*(Ibid.)*

5. *Effects of Iodine in Scrofula.*—The physician, whose case we have given above, has found the combination of iodine with mercury and tonics extremely useful in all very inveterate cases of scrofula, particularly in ill-grown children, with pale flaccid skin, and with the mental faculties but little developed, the belly large, the limbs crooked, the glands of the neck, axilla, and mesentery enlarged; in whom there existed, at the same time, a disposition to continual suppuration, without the discharge removing the glandular swellings; in whom the employment of internal and external remedies induces the sores to heal, but which soon break out again. In all such cases iodine, given alternately with calomel and tonics, produced great benefit. The tumors diminished in size, and finally disappeared; the suppuration in the glands ceased; the functions of the alimentary canal became reinstated, and the colour of the skin improved. Similar benefits resulted in local strumous affections, as ophthalmia and various eruptions. Dr. Kolley has frequently seen cases of this kind, of long standing, and hitherto regarded as incurable, permanently disappear under the use of iodine.

The diet recommended by this writer is analeptic, and taken from animal substances, but extremely rigid; particularly in cases of dartres, in which the patients are not allowed to take wine, spirits, nor even beer, but to drink abundance of water, and always to eat so little that a feeling of hunger remains; while they are to keep in a moderately elevated temperature.—*(Ibid.)*

6. *On the Action of the pure Meconic Acid, and its Combinations with Potash and Soda.*—A great difference of opinion having existed on the continent with regard to the action of the pure meconic acid, as well as the meconiates of potash and soda, on the animal economy, Dr. Fenoglio, together with N. Cesare and Donenico Blengini, of Turin, have performed several experiments, in order to discover the real power of these substances. It had been said that two persons had fallen victims to very minute doses (a grain) of the pure meconic acid, given as an anthelmintic; whilst, on the other hand, it had been taken by M. Suertuerner to the amount of five grains without inconvenience; and M. Sœmmering administered as much as ten grains of the pure acid, as well as the meconiate of soda, to dogs, without the least sensible effect. The experiments of Dr. Fenoglio prove distinctly that doses of eight grains of these salts, or of the pure acid, produce no deleterious effects on dogs, crows, or frogs; some of the animals experimented upon being suffered to live, and others afterwards destroyed by prussic acid. The same experiment was afterwards repeated on a horse; eight grains of the pure acid, of the meconiate of soda and of potash, being given separately, and at the distance of a few days from each other, to this animal, without any marked effect being produced. It only remained now to ascertain the effect of these substances in the human subject; and, on the 15th of May, 1822, the opportunity was offered of exhibiting them to two sisters afflicted with tænia. The first, twenty-one years of age, took four grains of meconiate of potash; the second, aged nineteen, took four grains of meconiate of soda. These medicines had no sensible effect; not producing the expulsion even of any portion of the worm, which was afterwards got rid of by Madame Nouffler's remedy.

In a foot-note annexed to this article, Dr. Fenoglio attributes, with every appearance of truth, the death of the two individuals above mentioned to the presence of morphia, from a defective preparation of the substance employed; and the account he gives of the symptoms that preceded their death gives great weight to his opinion.—*(Annali Universali*, November.)

### SURGERY.

7. *Removal of a Polypous Tumor from the Conjunctiva.*—Dr. Benaber, of Thoulouse, relates the case of a female, fifty-five years of age, and who had ceased to menstruate about six years, named Jane Merville. In the month of November, 1821, this woman perceived a soft and indolent wart growing from the outer angle of the right eye. This swelling was not at first larger than a lentil, and its growth was very slow, until the month of March, 1822, when it began to be painful, and the patient applied to an empyric, who applied some plasters to the eye; after which, the pains became more violent, and the tumor augmented rapidly. She then applied to a female practitioner, who applied burnt alum to the part, scarified it with a knife, and washed it with a decoction of some herbs. From that time the progress of the disease was frightfully rapid; the eye became concealed by a mushroom-shaped swelling, unequal, reddish in colour, and bleeding at the slightest touch. The patient applied to the Hôtel Dieu at Thoulouse in August, 1822; but her disease was considered incurable. The complaint now prevented sleep, and was attended with lancinating pains. She then sought the advice of Dr. Benaber, who found the tumor uneven on its surface, of a pale-red colour, about the size of a hen's egg, covering both the eyelids, and was distressing, as well by its weight as from the pain attending it; its consistence appeared hard, and yet it was torn easily. On raising a point of its circumference, the instrument penetrated it, and a light-coloured blood escaped. It appeared to have an outward membranous covering, about two lines in thickness; its interior was white, granulated, and full of red points. Dr. Benaber discovered that it had a pedicle, round which were numerous prolongations, having a structure analogous to the principal tumor, and which also arose from the conjunctiva. This membrane was greatly swollen; the lower lid was inverted.

On a minute examination, it appearing that the eye itself was sound, Dr. B. thought it advisable to tie the tumor: a flat ligature of several threads was therefore passed behind it, on the 5th of September, so as to enclose all the parts; and, although drawn but moderately tight, it caused violent pain, which however was much diminished the next day. To calm this irritation, a grain of opium was directed to be taken every evening; but the patient having got six pills, with one grain in each, took them *all* the same evening, without any ill consequence. The ligature was tightened from time to time, causing considerable pain; it gave way on the seventeenth day, in consequence of suppuration, and was replaced by another. At length, on the night of the thirtieth day, the tumor, which had shrunk considerably, fell off; and, in four days after, without the use of any other means, every appearance of disease was removed, excepting the eversion of the lower eyelid, which was remedied by the excision of several portions of the membrane covering it. There only remained a small loss of substance near the lacrymal point of the lower eyelid, causing a slight epiphora; but, although a year has elapsed, there is no sign of the reproduction of the tumor.—(*Revue Medicale,* December.)

---

### MIDWIFERY.

8. *Case of Malformation of the Fœtus.*—As the question, "Can the fœtus in utero be affected by any thing acting on the mother's imagination or feelings?" is one of great importance, and has never yet been satisfactorily answered, I take the liberty of sending you the description of a case, similar to that communicated by Mr. Morton, which occurred under my own observation a few years since, leaving my readers to draw their own inference.

Dec. 1, 1813, I attended a Mrs. G., who was delivered of a girl without arms, but in every other respect perfectly formed. From the shoulders there were projections of about two inches in length, and at the extremity of each a small thumb and finger, with perfect nails, but no appearance of a hand. In one of the projections a small bone could be felt, resembling in shape the os humeri. The infant lived a week; but, at its death, the mother could not be prevailed on to permit a scientific examination. She gave a similar account to that mentioned in Mr. Morton's narrative, of her having been alarmed, in an early stage of her pregnancy, by a sailor's suddenly exposing the stumps of his arms to her view, as a means of exciting her compassion and charity. While I readily agree with Mr. Morton, that, from anatomical considerations, it seems extremely improbable, if not impossible, that external circumstances, affecting the mother, should produce such extraordinary changes in the fœtus, the frequent occurrence of these phenomena, almost universally attributed to the agency of such circumstances, justify hesitation at least, and call for still further investigation.

The well-known story, told by Father MALBRANCHO, of the woman, in the second month of her pregnancy, who witnessed the breaking a criminal on the wheel, and felt herself terribly affected by the horrid sight, and afterwards gave birth to a child with limbs broken exactly like those of the culprit, is a strong case in favour of the generally-received opinion.—*(Private Letter from* Mr. HOARE, *Surgeon, Warminster.)*

---

### CHEMISTRY.

9. *New Alkali in Jalap.*—Mr. HUME, jun. of Long Acre, has just extracted from jalap a substance similar to the recently-discovered alkalies of opium, bark, nux vomica, &c. Having procured but a small quantity of this new substance, (for which he proposes the name of *jalapine,*) Mr. Hume is not yet enabled to give any satisfactory account of its chemical properties, nor of its action on the animal economy.

The mode of procuring jalapine is very simple. Coarsely-powdered jalap is macerated, for twelve or fourteen days, in strong acetic acid; a high-coloured tincture is thus obtained, which, when filtered, is to be supersaturated with ammonia, and the mixture violently shaken; a sabulous deposit will fall rapidly, and a few crystals will form on the sides of the vessel. The deposit and crystals are to be collected, and washed with distilled water; again dissolved in a small quantity of concentrated acetic acid, and reprecipitated by ammonia added to supersaturation, which throws down the jalapine, in small, white, acicular crystals.

Jalapine is without any perceptible taste or smell, and seems heavier than morphine, quinine, or other substances of this class; it is scarcely, if at all, soluble in cold water, and only to a small extent in hot water. Ether has no effect upon it; alcohol is its proper solvent. Very little trouble is requisite to purify jalapine from extractive or colouring matter, for which it appears to have a very slight affinity.

Mr. Hume, jun. operated only upon an ounce of jalap-root, from which quantity he conjectures, that, when the process is conducted with greater accuracy than usually attends a first examination, it will be easy to obtain about five grains of jalapine.—*(Private Letter from* Mr. HUME.)

---

### MISCELLANEOUS.

10. *Medical Society of London, Bolt-court.*—The fifty-first anniversary meeting of this Society was held at the London Coffee-house, Ludgate-hill, on Monday, March the 8th. The officers and council elected for the ensuing year are as follow:

*President*—Wm. Shearman, M.D.

*Vice-Presidents*—Henry Clutterbuck, M.D. Henry James Cholmely, M.D. Sir Astley Paston Cooper, Bart. F.R.S. and Thos. Callaway, Esq.

*Treasurer*—John Andree, Esq.

*Librarian*—David Uwins, M.D.

*Secretaries*—T. J. Pettigrew and Thomas Callaway, Esqrs.

*Secretary for Foreign Correspondence*—Robley Dunglison, Esq.

The other members of the *Council* are—Drs. Walshman, Hancock, J. G. Smith, Blicke, Blegborough, Hopkinson, Stewart, Ley, Darling, Haslam, Pierce, Cox, and Burne; Messrs. Sutliffe, Drysdale, Winder, Johnson, Dunlap, Kingdon, Ward, Burton, Brown, Thomas Clarke, Lake, Ashwell, Edwards, Handy, Leese, Skair, Cordell, Bell, Ellerby, Amesbury, Bryant, and Burrows.

*Registrar*—James Field, Esq.

*The Fellow elected to deliver the Anniversary Oration, in March* 1825—Eusebius Arthur Lloyd, Esq.

The President informed the meeting, that some of the notices inserted in the Medical Journals at the beginning of the year, having, by a mistake in copying, announced the 31st, instead of the 1st day of December, as the latest day for sending in the Dissertations for the Fothergillian medal, papers were received by the Society till the expiration of the latest period specified. The time allotted for the perusal of the Essays having thus been shortened, the Committee of Papers, appointed by the Council, had been under the necessity of postponing the adjudication of the medals for the Essays, written in the years 1822 and 1823, on the subjects of "Dropsy" and "Diseases of the Spine," till Monday the 3d day of May, at eight o'clock in the evening; at which time a special general meeting of the Society would be held.

The annual oration was then delivered by Dr. John Gordon Smith, the ex-Vice President: the subject was "the Duties and Perplexities of Medical Men, as professional Witnesses in Courts of Justice."

The Fellows and their friends, in number eighty-six, dined together in the great room of the tavern, the President being in the chair, and the evening was spent in the most harmonious and convivial manner.

*Conditions of the Fothergillian Medal.*—In conformity with the will of the late Dr. Anthony Fothergill, the Society resolve to give annually to the author of the best Dissertation on a subject proposed by them, a gold medal, value twenty guineas, called the *Fothergillian medal;* for which the learned of all countries are invited as candidates.

1. Each Dissertation must be delivered to the Registrar in the Latin or English Language, on or before the 1st day of December.

2. With each Dissertation must be delivered a sealed packet, with some motto or device on the outside, and within the author's name and designation; and the same motto or device must be put upon the Dissertation, that the Society may know how to address the successful candidate.

3. No paper in the hand-writing of the author, or with his name affixed, will be received; and if the author of any paper shall, directly or indirectly, discover himself to the Committee of Papers, or to any member thereof, such paper will be excluded from all competition for the medal.

4. All the Dissertations, the successful one excepted, will be returned, if desired, with the sealed packets unopened.

5. The prize medal will be presented to the successful candidate, or his substitute, at the anniversary meeting in March.

*** The subject of the Dissertation to be offered for the prize medal for 1825, is "The Pathology and Treatment of Periodical Asthma."

### METEOROLOGICAL JOURNAL,

*From February 20, to March 19, 1824.*

By Messrs. WILLIAM HARRIS and Co. 50, Holborn, London.

| | MOON. | Rain gauge | THERM. | | | BAROM. | | DE LUC'S HYG. | | WIND. | | ATMOSPHERIC VARIATION. | | |
|---|---|---|---|---|---|---|---|---|---|---|---|---|---|---|
| | | | 9 A.M. | MAX. | MIN. | 9 A.M. | 10P.M. | 9 A.M. | 10P.M. | 9 A.M. | 10P.M. | 9 A.M. | 2 P.M. | 10P.M. |
| Feb. | | | | | | | | | | | | | | |
| 20 | | .22 | 40 | 43 | 38 | 29.37 | 29.50 | 97 | 96 | ENE | NE | Rain | Rain | Rain |
| 21 | ☾ | | 37 | 41 | 31 | 29.63 | 29.72 | 94 | 97 | WSW | E | | Fair | Fair |
| 22 | | | 40 | 45 | 37 | 29.76 | 29.80 | 95 | 90 | ESE | SE | Foggy | Fine | Fine |
| 23 | | | 40 | 45 | 37 | 29.84 | 29.90 | 97 | 94 | ESE | E | Overc. | Fair | Fair |
| 24 | | | 40 | 43 | 37 | 29.84 | 29.73 | 90 | 85 | E | ESE | Fair | Fine | |
| 25 | | | 37 | 38 | 35 | 29.67 | 29.70 | 80 | 87 | ENE | NE | | Fair | |
| 26 | | | 36 | 37 | 34 | 29.67 | 29.60 | 92 | 96 | N | NE | Cloud. | | Sleet |
| 27 | | | 34 | 40 | 32 | 29.50 | 29.55 | 96 | 97 | NNE | E | Snow | Sleet | Rain |
| 28 | | | 37 | 40 | 35 | 29.68 | 29.76 | 98 | 98 | S | N | Foggy | Fair | |
| 29 | ● | $\frac{31}{53}$ | 39 | 40 | 35 | 29.76 | 29.76 | 98 | 95 | E | E | Rain | Overc. | Overc. |
| Mar | | | | | | | | | | | | | | |
| 1 | | | 39 | 40 | 30 | 29.63 | 29.50 | 80 | 89 | NW | N | Fine | Fine | Rain |
| 2 | | | 31 | 39 | 31 | 29.50 | 29.52 | 60 | 70 | N | NW | | | Fine |
| 3 | | | 38 | 40 | 29 | 28.93 | 29.61 | 90 | 85 | NW | N | Snow | Fine | Storm. |
| 4 | | | 29 | 40 | 33 | 29.70 | 29.70 | 70 | 75 | NNW | WSW | Fine | | Fine |
| 5 | | | 43 | 49 | 43 | 29.43 | 29.57 | 70 | 75 | NW | NW | Fair | Rain | Fair |
| 6 | | | 45 | 50 | 45 | 29.70 | 29.67 | 79 | 84 | SW | NW | Fine | Fine | |
| 7 | | | 48 | 50 | 45 | 29.40 | 29.40 | 98 | 98 | SW | W | Rain | Rain | Rain |
| 8 | ☽ | | 49 | 50 | 38 | 29.10 | 29.30 | 100 | 90 | SWva. | W | Storm. | | |
| 9 | | .50 | 40 | 45 | 39 | 29.60 | 29.54 | 67 | 67 | W | SSW | Fine | Fine | Fine |
| 10 | | | 40 | 45 | 33 | 29.54 | 29.76 | 85 | 85 | ENE | N | Foggy | Rain | Rain |
| 11 | | | 35 | 45 | 36 | 29.90 | 29.53 | 85 | 89 | NW | N W | Fine | | |
| 12 | | | 38 | 45 | 37 | 29.40 | 29.40 | 84 | 87 | W | W | Cloud. | Hail | Fair |
| 13 | | | 39 | 41 | 33 | 29.22 | 29.27 | 87 | 87 | W | WNW | Fair | | Fine |
| 14 | | | 40 | 45 | 35 | 29.72 | 29.90 | 72 | 75 | NE | W | Fine | Fine | |
| 15 | ○ | | 38 | 47 | 40 | 29.93 | 29.87 | 75 | 90 | SSW | NW | Fair | Fine | Rain |
| 16 | | | 40 | 50 | 42 | 29.84 | 29.85 | 95 | 90 | S | SW | Overc. | | Cloud. |
| 17 | | | 45 | 52 | 41 | 30.05 | 30.08 | 80 | 80 | W | NW | Fine | | Fine |
| 18 | | | 44 | 52 | 48 | 30.10 | 30.12 | 80 | 84 | W | WSW | | | |
| 19 | | .17 | 48 | 55 | 38 | 30.14 | 30.14 | 82 | 80 | W | NW | Overc. | Fair | |

The quantity of rain fallen in the month of February, was 53.100ths of an inch.

